# Hypertension treatment in sub-Saharan Africa: a systematic review

**DOI:** 10.5830/CVJA-2022-065

**Published:** 2023-05-25

**Authors:** Pauline Cavagna, Céline Leplay, Marie Antignac, Pauline Cavagna, Xavier Jouven, Marie Antignac, Roland N’Guetta, Kouadio Euloge Kramoh, Ibrahima Bara Diop, Dadhi M Balde, Jean Bruno Mipinda, Michel Azizi, Xavier Jouven, Michel Azizi, Xavier Jouven

**Affiliations:** Department of Pharmacy, Pitié Salpêtrière Hospital, AP-HP Sorbonne University, Paris, France; Université Paris Cité, Inserm, PARCC, Paris, France; Institute of Cardiology of Abidjan, Abidjan, Ivory Coast; Cardiology Department, University Hospital of Fann, Dakar, Senegal; Department of Cardiology, University Hospital of Conakry, Guinea; University Hospital of Libreville, Libreville, Gabon; Cardiovascular Epidemiology Department, University of Paris, Paris, France; Hypertension Unit, European Georges Pompidou Hospital, and National Institute of Health and Medical Research, Clinical Research Centre, Paris, France; Cardiology Department, European Georges Pompidou Hospital, Paris, France

**Keywords:** sub-Saharan Africa, developing countries, hypertension, antihypertensive medications

## Abstract

Sub-Saharan Africa (SSA) faces the highest rate of hypertension worldwide. Blood pressure (BP) control rests on the association of lifestyle modification and antihypertensive medicines. We aimed to systematically review antihypertensive strategies implemented in SSA to achieve BP control. A systematic search beginning in 2003 was performed in MEDLINE, COCHRANE and EMBASE. We included only original and observational studies in SSA countries. Thirty studies were included from 11 countries. No study was multinational. The number of patients varied from 111 to 897 (median: 294; IQR: 192–478). Overall, 21% of patients received monotherapy, 42.6% two-drug and 26.6% three-drug combinations. Out of all the strategies, renin–angiotensin system (RAS) blockers were mostly prescribed, followed by diuretics and calcium channel blockers. In monotherapy, RAS blockers were the first to be prescribed. Only 10 articles described antihypertensive strategies beyond triple combinations. BP control was highly variable (range: 16.4 to 61.2%). Multicentre studies performed in several SSA countries are needed to ensure international guidelines actually do improve outcomes in SSA.

In low- and middle-income countries, infectious and non-communicable diseases exist contemporarily.^1^ In those countries, recent studies have emphasised the increasing contribution of non-communicable diseases to the global burden of disease.^2^ The number of adults with raised blood pressure (BP) has increased alarmingly from 0.59 to 1.13 billion between 1975 and 2015, especially in low- and middle-income countries.^3^ Furthermore, epidemiological trends show that the burden of hypertension in Sub-Saharan Africa (SSA) has overtaken that of many European and North American states.^3^ The highest prevalence of hypertension is found in SSA, with a three-fold higher mortality rate from stroke and other associated disease.^4^,^5^

The association of lifestyle measures and antihypertensive medicines are the cornerstone of hypertension control.^6^ Randomised clinical trials conducted in high-income countries have proven that antihypertensive medication therapy reduces BP and cardiovascular, cerebrovascular and renal morbidity and mortality rates.^7^ The World Health Organisation (WHO) aims to achieve a 25% relative reduction in the prevalence of raised BP in SSA in 2025.^4^

Both European and USA guidelines contain specific recommendations for the treatment of black hypertensive adults,^6^,^8^ since it is well established that blacks living in the Northern Hemisphere respond differently to antihypertensive agents compared to white hypertensive adults due to a variety of phenotypic differences.^9^ However, studies in African-Americans or African-Caribbeans are not necessarily generalisable to SSA populations due to both contrasting environmental settings and genetic diversity.^10^

There have been a few comprehensive syntheses of pharmacotherapy used to manage hypertension in SSA.^5^ We aimed to systematically review antihypertensive strategies implemented in SSA to achieve BP control in real clinical practice.

## Methods

This systematic review was registered in the PROSPERO database (CR42019146769) and was reported according to the Preferred Reporting Items for Systematic Reviews and Meta- Analyses (PRISMA) guidelines.^11^

We included original studies describing how antihypertensive medications were prescribed in SSA countries and assessing the influence of the different strategies on BP control. We considered only observational studies in patients over 18 years with primary hypertension and living in SAA. We excluded randomised, control trials. Studies where the principal inclusion criterion was not hypertension (such as eclampsia, stroke, HIV, cancer, infections, asthma, diabetes mellitus, neurovascular disease or pulmonary hypertension) were excluded.

We searched MEDLINE via PUBMED, COCHRANE and EMBASE using a strategy relying on algorithms adapted to each database, including specific key words (MeSH terms for MEDLINE and Emtree terms for EMBASE) and free text words (Table 1). The electronic research was run on 25 April 2020 without language restrictions. We restricted our search to begin in 2003 because it was the year of publication of guidelines on the management of hypertension by ethnicity in major worldwide savant society.

**Table 1:** Search algorithms in three databases

*# Search*	Results
Search algorithms and results in MEDLINE	
#1 ((((((Therapeutics[Title/Abstract]) OR Therapy[Title/Abstract]) OR Treatment[Title/Abstract]) OR Drug Therapy[Title/Abstract]) OR Medicines[Title/ Abstract]) OR Antihypertensive agents[MeSH Terms])	
#2 ((Hypertension[Title/Abstract]) OR High blood pressure[MeSH Terms])	
#3 (((((((Sub-saharan Africa[MeSH Terms]) OR Black people(Title/Abstract]) OR middle income countries[Title/Abstract]) OR low income countries[Title/ Abstract]) OR poor countries[Title/Abstract]) OR developing countries[Title/Abstract])OR Africa south of the sahara[Title/Abstract]	
#4 #1 AND #2 AND #3	1817
Search algorithms and results in EMBASE	
#1 'africa south of the sahara'/exp OR 'sub-saharan africa':ab,ti OR 'black people':ab,ti OR 'black person':ab,ti OR 'middle income countries':ab,ti OR 'low income countries':ab,ti OR 'poor countries':ab,ti OR 'developing country':ab,ti	
#2 therapeutics:ab,t OR therapy:ab,ti OR treatment:ab,ti OR 'drug therapy':ab,ti OR medicines:ab,ti OR "antihypertensive agents'/exp	
#3 'hypertension'/exp OR 'high blood pressure':ab,ti	
#4 #1 AND #2 AND #3	2352
Search algorithms and results in Cochrane Central Register of Controlled Trials	
#1 MeSH descriptor: [Antihypertensive agent] explode all trees	
#2 MeSH descriptor: [Hypertension] explode all trees	
#3 (MeSH descriptor: [Africa South of the Sahara] explode all trees	
#4 (antihypertensive agents):ti,ab,kw OR (drug therapy):ti,ab,kw OR (therapeutics):ti,ab,kw OR (medecines):ti,ab,kw OR (treatments):ti,ab,kw	
#5 (hypertension): ti,ab,kw OR (high blood pressure): ti,ab,kw	
#6 (sub-Saharan Africa): ti,ab,kw OR (low income countries): ti,ab,kw OR (middle income countries): ti,ab,kw OR (black people): ti,ab,kw OR (developing countries):ti,ab,kw	
#7 (#1 OR #4) AND (#2 OR #5) AND (#3 OR #6)	444

exp: explosion, ti: title, ab: abstract

After removing duplicates, one investigator (PC) screened the titles and abstracts. Then, two investigators (PC, CL) screened full texts independently to identify eligible articles. Disagreements were resolved by discussion with the help of a third investigator (MAN) whenever necessary, to reach consensus.

Data extraction was independently performed using a standardised form by two authors (PC, CL). The list of data items collected for each included study was as follows:

- General information: first author, affiliation of the first and the last author, year of publication, journal, country, study design, study duration, whether the study was single or multicentre, whether it was prospective or retrospective, inclusion and exclusion criteria, and sample size.
- Baseline population characteristics: number of patients, mean age, gender ratio, co-morbidities, cardiovascular risk factors, individual wealth index, and patient location (urban or rural).
- Hypertension and treatment: duration of hypertension, mean office systolic and diastolic BP value before and after treatment, prescribed antihypertensive medication classes, proportion of adherent patients, proportion of patients with controlled hypertension, and severity of hypertension
- Guideline use to define hypertension control and severity, and treatment.

Each country’s income was obtained from the World Bank database,^12^ (accessed 2 February 2020) and categorised into low-income, lower middle-income and upper middle-income countries.

We used the National Institute of Health (NIH) quality assessment tools for risk of bias assessment.^13^ This tool was preferred because it is more comprehensive and therefore enables an exhaustive assessment of quality of included studies. The overall quality of included studies was rated as good, fair and poor.

The quality of reporting was assessed for each included study according to the STROBE statement.^14^ Each item was coded as completely reported, not completely reported or not reported.

These assessments (risk of bias and quality of reporting) were performed independently by PC and CL with the help of MAN in the case of disagreement.

## Statistical analysis

Agreement between the two investigators for study eligibility was assessed by the Cohen kappa coefficient: a kappa coefficient = 0.60–0.74 was considered good, and ≥ 0.75 was very good.

Data were expressed as mean or percentage when available. Median and interquartile range were calculated. Analyses were performed through script developed in the R software [version 3.5.1 (2018–07–02)].

## Results

**Fig. 1:**
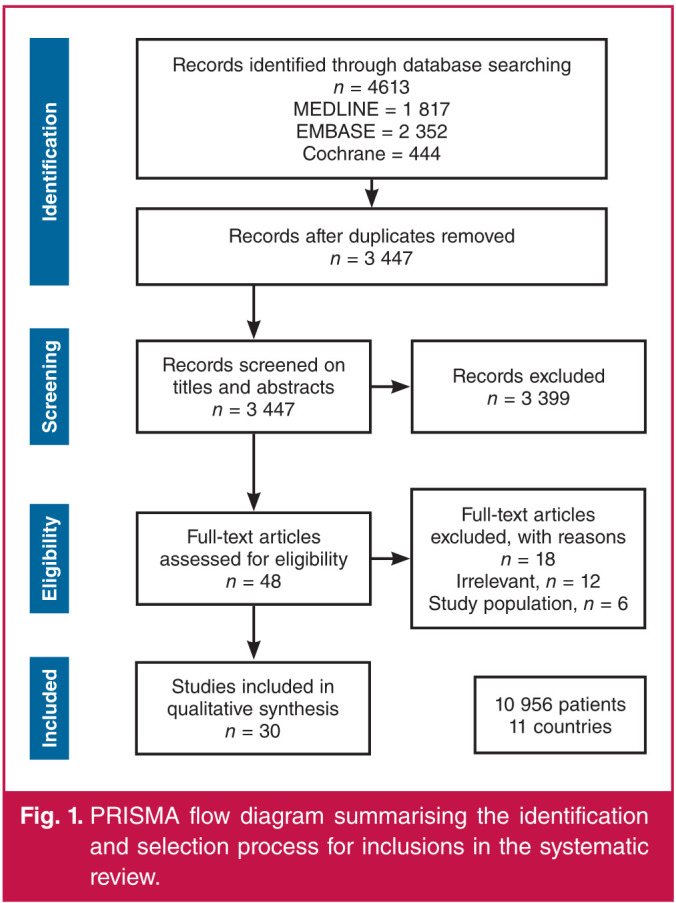
PRISMA flow diagram summarising the identification and selection process for inclusions in the systematic review.

We identified 4 613 references, resulting in 3 447 unique citations, after removing duplicates. A flow diagram summarising the identification and selection process for included studies is presented in Fig. 1. We screened 3 447 studies on the basis of the titles and abstracts leading to the exclusion of 3 399 irrelevant citations. A total of 48 publications were reviewed for eligibility on the basis of full-text reading. Thirty studies10,^15^–43 met the eligibility criteria. Agreement for full-text eligibility between the two investigators was very good [Cohen kappa coefficient 0.75 (0.55–0.96)].

**Fig. 2:**
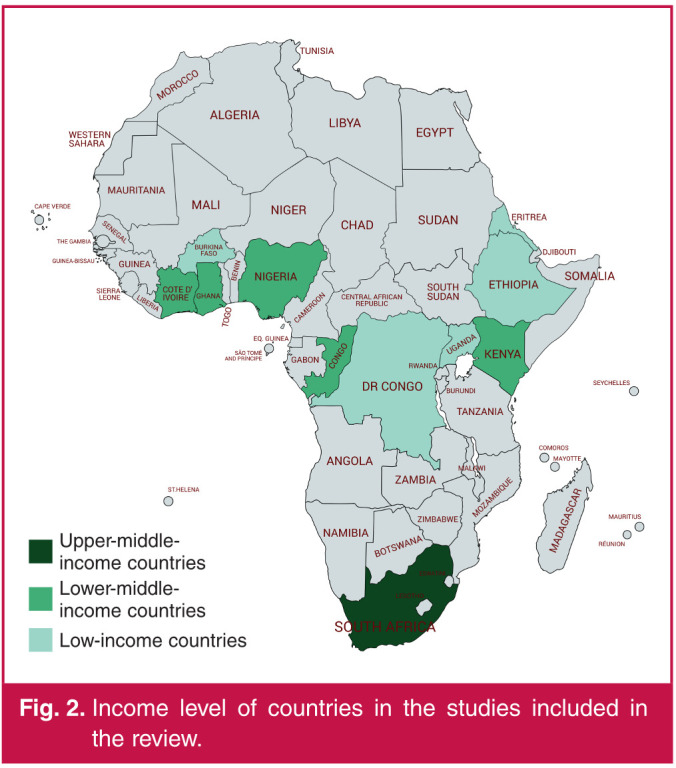
Income level of countries in the studies included in the review.

**Fig. 3:**
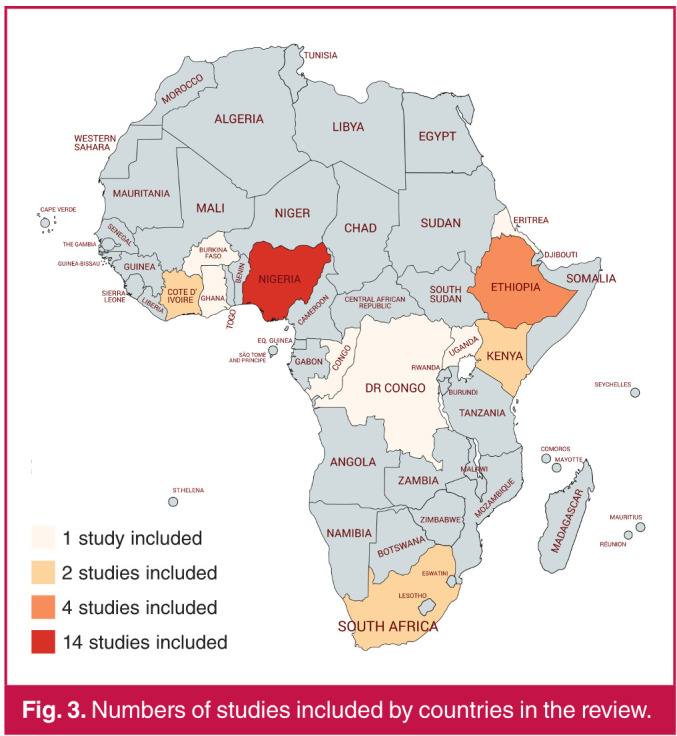
Numbers of studies included by countries in the review.

The study characteristics are presented in Table 2. The 30 studies from 11 countries were published between 2003 and 2018. Among the 11 countries in the review, five countries were low income (Burkina Faso, Democratic Republic of the Congo, Uganda, Ethiopia, Eritrea), five were lower middle income (Cote d’Ivoire, Ghana, Nigeria, Congo, Kenya) and one country was upper middle income (South Africa) (Fig. 2). Most of the studies were cross-sectional studies (21 articles). Three studies were multicentre19,^20^,^40^ and none was multinational. Almost half of the studies were conducted in Nigeria (14 articles) (Fig. 3), four were conducted in Ethiopia, two in Cote d’Ivoire and two in South Africa. The other eight studies were conducted in various countries of SSA. Almost all studies unfolded in tertiary-carecentres (27 articles). Antihypertensive medications were never provided free of charge to patients.

**Table 2:** Summary of study characteristics

*First author*	*Year*	*Study design 1*	*Study design 2*	*No. of countries*	*Country*	*No. of centres*	*Type of centres*	*No. of patients*	*Definition of hyper- tension*	*Definition of severity of hyper- tension*	Drugs provided
Adigun et al.	2003	Cross-sectional	Prospective	1	Nigeria	1	Tertiary care	150	Yes	-	No
Hesse et al.	2013	-	Retrospective	1	Ghana	1	Tertiary care	155	Yes	-	No
Yusuff et al.	2004	Cross-sectional	Retrospective	1	Nigeria	1	Out-patient tertiary care	189	Yes	Yes	No
Yusuff et al.	2005	Cross-sectional	Retrospective	1	Nigeria	1	Out-patient tertiary care	189	Yes	Yes	No
Etuk et al.	2008	Cross-sectional	Retrospective	1	Nigeria	1	Tertiary care	145	Yes	Yes	No
Pillay et al.	2009	-	-	1	South Africa	16	Tertiary care	-	-	-	No
Rayner et al.	2009	Cross-sectional	-	1	South Africa	15	Primary care	451	Yes	-	No
Olanrewaju et al.	2010	Cross-sectional	-	1	Nigeria	1	Tertiary care	787	-	-	No
Ganiyu et al.	2012	-	Retrospective	1	Nigeria	1	Tertiary care	208	Yes	Yes	No
Ilesanmi et al.	2012	Cross-sectional	-	1	Nigeria	1	Tertiary care	250	Yes	Yes	No
Konin et al.	2011	Cross-sectional	Prospective	1	Côte d'Ivoire	-	Tertiary care	144	Yes	-	No
Kramoh et al.	2011	-	Retrospective	1	Côte d'Ivoire	1	Tertiary care	854	Yes	Yes	No
Omole et al.	2011	Cohort study	Retrospective	1	Nigeria	1	Tertiary care	230	Yes	Yes	No
Tamuno et al.	2012	Cross-sectional	Retrospective	1	Nigeria	1	Tertiary care	200	Yes	Yes	No
Ukwe et al.	2012	Cross-sectional	Retrospective	1	Nigeria	1	Out-patient tertiary care	376	Yes	Yes	No
Ojji et al.	2013	-	-	1	Nigeria	1	Tertiary care	590	-	-	No
Shobana et al.	2013	-	Retrospective	1	Eritrea	1	Tertiary care	111	Yes	Yes	No
Yaméogo et al.	2012	Cross-sectional	-	1	Burkina Faso	1	Out-patient tertiary care	456	Yes	-	No
Mutua et al.	2014	Cross-sectional	-	1	Kenya	1	Out-patient tertiary care	452	Yes	Yes	No
Ikama et al.	2015	Cross-sectional	Prospective	1	Congo	1	Tertiary care	620	Yes	-	No
Shukrala et al.	2015	Cross-sectional	Prospective Retrospective	1	Ethiopia	1	Out-patient tertiary care	400	Yes	Yes	No
Kika et al.	2016	Cross-sectional	-	1	Democratic Republic of the Congo	1	Primary care	298	Yes	-	No
Ssianulya et al.	2016	-	Retrospective	1	Uganda	1	Tertiary care	741	Yes	-	No
Adejumo et al.	2017	Cross-sectional	-	1	Nigeria	1	Out-patient tertiary care	224	Yes	-	No
Berhe et al.	2017	Cohort study	Retrospective	1	Ethiopia	6	Out-patient tertiary care	897	Yes	Yes	No
Mbui et al.	2017	Cross-sectional	Retrospective	1	Kenya	1	Out-patient tertiary care	247	Yes	Yes	No
Olowofela et al.	2017	Cross-sectional	-	1	Nigeria	1	Out-patient tertiary care	514	Yes	-	No
Teshome et al.	2018	Cross-sectional	-	1	Ethiopia	1	Out-patient tertiary care	392	Yes	-	No

Of the 30 articles included, 29 (96.6%) had at least one author affiliated to an African institution. Almost all of the articles reviewed had a first author affiliated to an African institution (93.3%), while 86.6% had a last author affiliated to an African institution. Only three articles40,^41^,^43^ had one first or last African author with an affiliation from an English-speaking institution.

The patients’ characteristics are displayed in the Table 3. Among the articles reviewed, the number of patients included varied from 111 to 897, with a median by study of 294 (IQR: 192–478). Among the 10 456 patients studied overall, 40.7% (n = 4 252) were Nigerian patients. The median patient age was 58 years (IQR: 54–61). The median gender ratio (M:F) was 0.7 (IQR: 0.5–0.9). The duration of hypertension varied widely and was reported in eight articles.^10^,^25^,^30^,^31^,^33^,^37^,^39^,^42^ Severity of hypertension was reported in 14 articles. Among them, proportion of patients with hypertension grade 1, 2 and 3 was 27.5% (IQR: 17.6–30.3%), 29.4% (IQR: 29–31%) and 32.3% (IQR: 17.3–34.1%), respectively (Table 4).

**Table 3:** Patients’ characteristics

*First author*	*No. of patients*	*Average age*	*MIF ratio*	*Diabetes mellitus (%)*	*Cardio- vascular disease history (%)*	*Obesity (%)*	*Dyslipi- daemia (%)*	*Renal failure (%)*	*Tobacco use (%)*	*Hypercho- lesterolae- mia (%)*	*Cardio- vascular family history (%)*	Sedentary lifestyle (%)	Metabolic syndrome (%)	Hypertri- glyceri- daemia (%)
Adigun et al.	150	61	0.9	21	-	-	-	-	-	-	-	-		
Hesse et al.	155	55	0.3	-	-	-	-	-	-	-	-	-	-	
Yusuff et al.	189	-	-	39.6	-	-	-	6.3	-	-	-	-	-	
Yusuff et al.	189	-	-	32.7	9.1	-	-	7.3	-	-	-	-	-	
Etuk et al.	145	52.3	0.9	-	-	-	-	-	-	-	-	-	-	
Pillay et al.	-	-	-	-	-	-	-	-	-	-	-	-	-	
Rayner et al.	451	60.7	0.8	18.6	-	36.4	-	-	15.7	44.3	-	-	17.1	
Olanrewaju et al.	787	57.8	0.6		-	-	-	-	-	-	-	-	-	
Ganiyu et al.	208	-	1.5	13.9	21.2	-	5.3	-	-	-	-	-	-	
Ilesanmi et al.	250	61	0.7	-	-	-	-	-	-	-	-	-	-	
Konin et al.	144	43.8	0.7	3.47	-	20.8	-	-	6.25	13.2	-	-	-	
Kramoh et al.	854	73.1	0.7	11.5	-	33.80	56	-	23.70	-	-	-	-	
Omole et al.	230	51	1.4	7	4.4	-	-	29.6	-	-	-	-	-	
Tamuno et al.	200	50.7	0.7	13	3.50	6.50	7.50	6.5	-	-	-	-	-	
Ukwe et al.	376	61	1.0	19.7	71	4.87	1.54	-	-	-	-	-	-	
Ojji et al.	590	49.7	1.0	-	-	-	-	-	-	-	-	-	-	
Shoba- et al.	111	58.3	1.7		100	-	-	-	-	-	-	-	-	
Yaméogo et al.	456	-	0.8	24.5	-	21	30	-	7	-	-	11	-	
Mutua et al.	452	63	0.4	41.8	4	-	-	-	-	-	-	-	-	
Ikama et al.	620	53.8	1.3	14.5	-	21.6	19.5	-	4	-	28	44.4	-	
Shukrala et al.	400	-	0.6	64.3	17.4	-	-	-	-	-	-	-	-	
Bakare et al.	200	58.4	0.5	11	41	5	7.5	-	-	-	-	-	-	
Busser et al.	486	-	0.8	-	-	-	-	-	-	-	-	-	-	
Kika et al.	298	64	0.4	37	-	17	-	30	3	17	50	-	10	18
Ssianulya et al.	741	-	0.2	1.5	1.9	-	0.5	-	-	-	-	-	-	
Adejumo et al.	224	59.6	0.5	10.7	-	-	-	-	-	-	-	-	-	
Berhe et al.	897	57	0.6	25	7	-	5	3	6	-	-	-	-	
Mbui et al.	247	55.8	0.1	36.8	2	-	-	-	-	-	-	-	-	
Olowofela et al.	514	57.9	0.5	25.5%	-	3.1	-	-	3.1	-	-	-	-	
Teshome et al.	392	58	0.9	18.4	17.3	-	-	6.6	1.3	-	20.7	-	-	
Median	274	58	0.7	18.6	9.1	17	6.4	6.6	6	17	28	27.7	13.5	18
IQR1-IQR3	192-478	54-61	0.5-0.9	11.2-29.1	4-21.2	5-21	4.1-13.1	6.4-18.4	3.1-7	15.1-30.6	24.3-39	19.3-36	11.7-15.3	
Min-max	111-897	43.8-6]	0.3-1.7	3.47-64.3	2-100	3.1-36.4	0.5-56	3-30	1.3-23.7	13.2-44.3	20.7-50	11-44.4	10-17.1	

**Table 4:** Main results of interest from the systematic review

*First Author*	*Mean of SBPI DBP*	*Controlled patients (%)*	*Grade 1 (%)*	*Grade 2 (%)*	*Grade 3 (#)*	*Lifestyle measures*	*Mono- therapy (%)*	*Two- drug strategies (%)*	*Three- drug strategies (%)*	*Single pill (%)*	*CCB (%)*	*Diuretic (%)*	*ACEI (%)*	*ARB (%)*	*RAS blockers*	*Beta- blockers*	*Centrally active drugs*	*Vasodilators*
Adigun et al.		47			17.3	No	39	52	8.7	7	51	56	24			5	28	
Hesse et al.	-	26		-		No	14	64	22.0	-	-			-	-	-		
Yusuff et al.	-	33.9	27.5	27	33.3	No	-		-	-	21	39.4	8.6	-	-	1.9	23.3	
Yusuff et al.	-	29	27.5	29	34.1	No	27	52.3	18	-	-			-	-	-		
Etuk et al.	-	30.5	29	29	35.9	No	20	48.6	26.9					-	-			
Pillay et al.	-	-	-	-	-	No	9-49	-	-		24.2	85	57.6	-	-	13.1	9.5	
Rayner et al.		61.2		-	-	Yes	30.7	42.8										
Olanrewaju et al.	-					No	9.1	37.1	35.8		65.8	84.4	66	5		12.7	28.5	
Ganiyu	132.2	16.4				Yes		36.7	41.8,		21,	49.8,	10.3,	0.8,		8.4,	9.1,	0,
et al.	18.6/84.9 + 12.1								41.3		21.8	46.9	11.7	4.4		8.7	5.6	0.2
Ilesanmi et al.		33.6	17.6	82.4		No	12.8	62.8	23.2		32	87.2	16.8			2	73.6	
Konin et al.		44.6,47.1, 47.9				Yes	49.6	36.4								-		
Kramoh	169.4 +	42.6	4.8	14.7	32.3	No	33.1	53.8	11		31.6	63.5			61.3	19	4.5	
et al.	28.4/95.3 + 15.7																	
Omole et al.		46		-		No	21.3		5.70	-	22	34.7	20.9	-		5	15.8	0.5
Tamuno et al.		34.50	21.5	29.5	42	Yes	8.5	42.5	30.5	7.5	22.8	34	23.9	4.60		-	6	
Ukwe et al.	163/99 and 141/87	3.5,18.9	11.4	45.5,15.4	-	Yes	27.6, 9.3	48.9,	38.220.4, 34.5		15.1, 15.9	48.2 and 46.4	18.1, 24.6	0.1,	18.2,25.6	3, 2.6	7.5,8.7	
Ojji et al.	148/93					No	5	28	31	17	67	54	48	10.7		34	5	5
Shobana et al.			34.2	59.4		No	27.9	45	27			72	54			72.9		
Yaméogo et al.		45.8				Yes	14.6	-	-		38.6	63.6	67.3	19.7		16.4		
Mutua et al.		33.40	62.5% of	37.5% of		No	16.6	42.3			37.1	78	53	21.7		36.1		
			uncon- trolled BP	uncon- trolled BP														
Ikama et al.	139/88.2	34.7		-		No	21	46.3	24.8									
Shukrala et al.		-	69	31		No	65	34	1	-	4.6	55	22.3	-		6.9	11.2	
Bakare et al.	130.6/80		49.5	38.50		No	2.5	28.5	36.5	1	53	64	52	17		63		
Busser et al.			30.3	29.4	12.3	Yes	43	48.7	7.8	-	-	-	-	-	-	-		
Kika et al.	151/87	22.5		-	-	No	66	34	0.0	17				-				
Ssianulya et al.		26.7		-		No	5.8	32.8	42.2		72.3	77.1			72.7	52.2	4.9	
Adejumo et al.	-	53.6				No	17.8	49.6		51.8	54.9	64.7	44.6	27.7		20.5	9.4	
Berhe et al.	-	37			-	No	38,4	45	55	-	50, 49%	56, 54%	56,55	-	-	19,19	-	
Mbui et al.	141/83	46	46.5, 34.8	42.5, 19	-	No	40	44	16	-	26	45.3	48.2	27.1	-	28.7	0.8	
Olowofela et al.		-			-	No	13	27.6	26.6	-	70.4	53	54		-	23	18.4	
Teshome et al.	142/87.7	42.9				Yes	58	39.2	2.8	-						-		
Median	145/87	33.9	27.5	29.4	32.3		21	42.6	26.6	12	34.5	59.7	48	13.8	67	19	9.4	0.5
IQR1- IQR3	141/84 154/93	29-43	17.6-30.3	29-31	17.3-34.1		12.9-39.5	35.8-49	8.7-36.5	7-17	24-58	48-76	22.4-53.5	4.9-23	64-69	6.8-31.3	[5.1-22]	0.3-2.7
Min-max	169.4 28.4/95.3 + 15.7	16.4-61.2	4.8-69	14.7-59.4	12.3-42		5-66	27.6-64	0-55	1-51.8	4.6-72.3	34-87.2	8.6-67.3		0.1-27.718.2-72.7	2-72.9	0.8-73.6	0-5

Among the patients’ cardiovascular risk factors, diabetes mellitus was the most common co-morbidity reported (22 articles). Cardiovascular history was the second most common cardiovascular risk factor reported (13 articles), followed by obesity (10 articles), dyslipidaemia (nine articles), renal failure (seven articles), cardiovascular family history (three articles) or sedentary lifestyle (two articles). There was large betweenstudy variability of cardiovascular risk factors associated with hypertension. The proportion of patients suffering from diabetes mellitus ranged from 3.47 to 64.3%, with a median of 18.6% (IQR: 11.2–29.1%). The proportion of patients with cardiovascular history varied from two to 100%, with a median of 9.1% (IQR: 4–21.2%). The proportion of obese patients varied from 3.1 to 36.4%, with a median of 17% (IQR: 5–21%). The proportion of patients with dyslipidaemia varied from 0.5 to 56%, with a median of 6.4% (IQR: 4.1–13.1%).

**Fig. 4:**
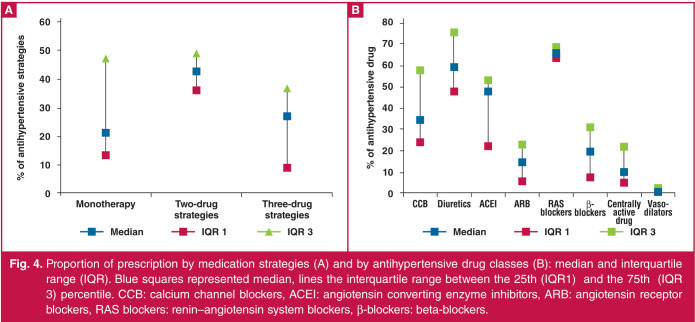
Proportion of prescription by medication strategies (A) and by antihypertensive drug classes (B): median and interquartile range (IQR). Blue squares represented median, lines the interquartile range between the 25th (IQR1) and the 75th (IQR 3) percentile. CCB: calcium channel blockers, ACEI: angiotensin converting enzyme inhibitors, ARB: angiotensin receptor blockers, RAS blockers: renin–angiotensin system blockers, β-blockers: beta-blockers.

Antihypertensive treatment strategies used in SSA countries and hypertension characteristics of patients are displayed in Table 4. Antihypertensive treatment strategies were described in 29 articles. There were some missing data for monotherapies (two articles), two-drug strategies (three articles) and threedrug strategies (seven articles). However, 10 articles described antihypertensive drug strategies beyond three-drug strategies. Overall, 21% (IQR: 12.9–39.5%) of patients received monotherapy, 42.6% (IQR: 35.8–49%) a two-drug combination and 26.6% (IQR: 8.7–36.5%) a three-drug combination (Fig 4A). Prescription of a single-pill combination was described in six articles (20%). According to these studies, one to 51.8% of patients received single-pill combination therapy.

Among all the treatment strategies, antihypertensive medication classes prescribed were not systematically described (21 articles; 70%). Angiotensin converting enzyme inhibitors (ACEI) and angiotensin receptor blockers (ARB) were not systematically differentiated and were combined under the common class of renin–angiotensin system (RAS) blockers in three articles.^25^,^28^,^38^ RAS blockers were the most commonly prescribed BP-lowering drugs [total RAS blockers: 67% (IQR: 64–69%); ACEI only: 48% (IQR: 22.4–53.5%); ARB only: 13.8% (IQR: 4.9–23%)], followed by diuretics (median: 59.7%; IQR: 48–76%) (Fig 4B).

**Fig. 5:**
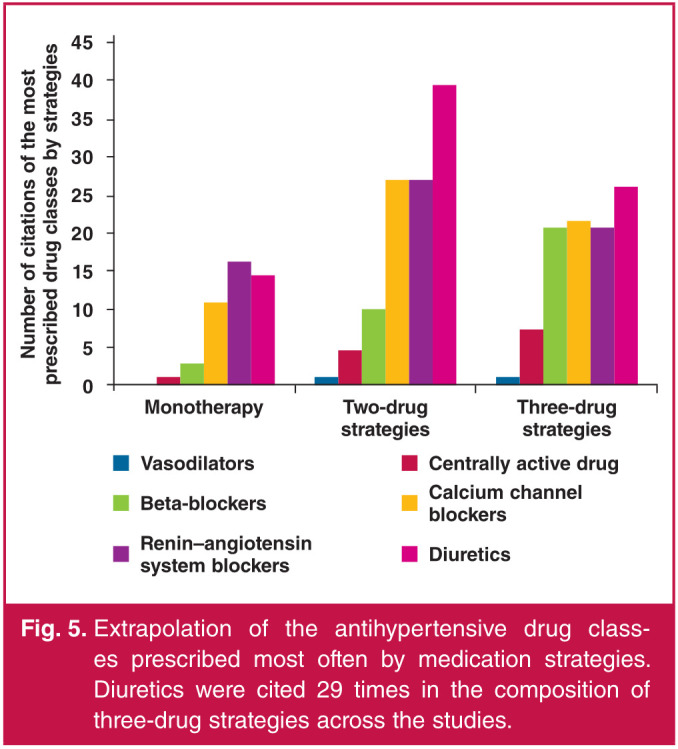
Extrapolation of the antihypertensive drug classes prescribed most often by medication strategies. Diuretics were cited 29 times in the composition of three-drug strategies across the studies.

Calcium-channel blockers (CCB) were the third antihypertensive drug class mostly prescribed, with a median of prescription of 34.5% (IQR: 24–58%). RAS blockers were the most widely cited drugs in monotherapy, followed by CCB (Fig. 5). Diuretics were the most common drugs cited in two-drug combinations, followed by RAS blockers and CCB. Beta-blockers were most frequently cited as part of a threedrug antihypertensive medication strategy, followed by RAS blockers. Antihypertensive medications by drug strategies were not itemised by quantitative data.

Of the 30 articles, nine described adherence to medication.^17^,^18^,^24^,^27^,^28^,^31^,^37^,^40^,^43^ Methods to assess adherence were described in three articles and based on questionnaires filled in by patients.^24^,^40^,^43^ For the other six studies, the physicians assessed adherence without clear methodology. A total of eight articles (26%) described combination of lifestyle measures and antihypertensive medications.

Of the 30 articles, six (20 %) did not report the proportion of patients with BP control or average office BP measurements before and after treatment. Hypertension was often defined (27 studies) but guidelines used to define hypertension and threshold of BP control were not systematically described (19 studies). Different guidelines were used across the studies (US Joint National Committee,^44^–46 European Society of Cardiology^47^,^48^ or WHO).^49^ Proportions of studies describing BP control, BP office measurements or both were 50% (n = 15); 6.6% (n = 2) and 23.3% (n = 7), respectively. The proportion of patients with office BP control was highly variable, ranging from 16.4 to 61.2%, with a median of 33.3% (IQR: 29–43%; Table 4). BP control was described globally but not by drug strategies.

Regarding risk of bias assessment (Table 5), one study had an overall risk of bias rating of good.^40^ Six studies were rated poor,^20^,^21^,^26^,^30^,^36^,^38^ and the remaining 23 articles were rated fair. In general, studies lacked sample size justification and some studies did not clearly and consistently define or implement the exposure measures across all study participants. Only six studies^31^,^32^,^37^,^40^,^41^,^43^ did adjust for key potential confounding variables. In addition, some items of the quality assessment tools were not reported across the studies.

**Table 5:** Risk-of-bias assessment

	CK1	CK 2	CK 3	CK 4	CK 5	CK6	CK 7	CK 8	CK 9	CK 10	CK 11	CK 12	CK 13	CK 14	Quality
Adigun et al.	Yes	Yes	NR	Yes	No	No	No	NA	Yes	No	Yes	NA	NA	No	Fair
Hesse et al.	Yes	Yes	NR	Yes	No	No	No	NA	Yes	Yes	Yes	NA	NR	No	Fair
Yusuff et al.	Yes	Yes	NR	Yes	No	No	No	NA	Yes	No	Yes	NA	NA	No	Fair
Yusuff et al.	Yes	Yes	NR	Yes	No	No	No	NA	Yes	No	Yes	NA	NA	No	Fair
Etuk et al.	Yes	Yes	NR	Yes	No	No	No	NA	Yes	No	Yes	NA	NA	No	Fair
Pillay et al.	Yes	Yes	NR	No	Yes	No	No	NA	No	Yes	Yes	NA	NA	No	Fair
Rayner et al.	Yes	Yes	NR	No	Yes	No	No	NA	No	No	Yes	NA	NA	No	Poor
Olanrewaju et al.	Yes	Yes	NR	Yes	No	No	No	NA	No	No	Yes	NA	NA	No	Poor
Ga et al. niyu	Yes	Yes	NR	Yes	No	No	No	NA	Yes	No	Yes	NA	NA	No	Fair
Ilesanmi et al.	Yes	Yes	NR	Yes	No	No	No	NA	Yes	No	Yes	NA	NA	No	Fair
Konin et al.	Yes	Yes	NR	Yes	No	No	No	NA	Yes	No	Yes	NA	NA	No	Fair
Kramoh et al.	Yes	Yes	NR	Yes	No	No	No	NA	Yes	Yes	Yes	NA	NA	No	Fair
Omole et al.	Yes	Yes	NR	Yes	No	No	No	NA	No	No	Yes	NA	NA	No	Poor
Tamuno et al.	Yes	Yes	NR	Yes	No	No	No	NA	Yes	No	Yes	NA	NA	No	Fair
Ukwe et al.	Yes	Yes	NR	Yes	No	No	No	NA	Yes	No	Yes	NA	NA	No	Fair
Ojji et al.	Yes	Yes	NR	Yes	No	No	No	NA	Yes	No	Yes	NA	NA	No	Fair
Shoba- et al.	Yes	Yes	NR	Yes	No	No	No	NA	No	No	Yes	NA	NA	No	Poor
Yaméogo et al.	Yes	Yes	NR	Yes	No	No	No	NA	Yes	No	Yes	NA	NA	Yes	Fair
Mutua et al.	Yes	Yes	NR	Yes	Yes	No	No	NA	Yes	No	Yes	NA	NA	Yes	Fair
Ikama et al.	Yes	Yes	NR	Yes	No	No	No	NA	Yes	No	Yes	NA	NA	NR	Fair
Shukrala et al.	Yes	Yes	NR	Yes	No	No	No	NA	Yes	No	Yes	NA	NA	No	Fair
Bakare et al.	Yes	Yes	NR	Yes	No	No	No	NA	Yes	No	Yes	NA	NA	No	Fair
Busser et al.	Yes	Yes	NR	Yes	No	No	No	NA	No	No	Yes	NA	NA	No	Poor
Kika et al.	Yes	Yes	NR	Yes	No	No	No	NA	Yes	No	Yes	NA	NA	Yes	Fair
Ssianulya et al.	Yes	Yes	NR	Yes	No	No	No	NA	No	No	Yes	NA	NA	No	Poor
Adejumo et al.	Yes	Yes	NR	Yes	Yes	No	No	NA	Yes	No	Yes	NA	NA	No	Fair
Berhe et al.	Yes	Yes	NR	Yes	Yes	No	No	NA	Yes	Yes	Yes	NA	Yes	Yes	Good
Mbui et al.	Yes	Yes	No	Yes	Yes	No	No	NA	Yes	No	Yes	NA	NA	Yes	Fair
Olowofela et al.	Yes	Yes	NR	Yes	Yes	No	No	NA	Yes	No	Yes	NA	NA	No	Fair
Teshome et al.	Yes	Yes	NR	Yes	Yes	No	No	NA	Yes	No	Yes	NA	NA	Yes	Fair

**Fig. 6:**
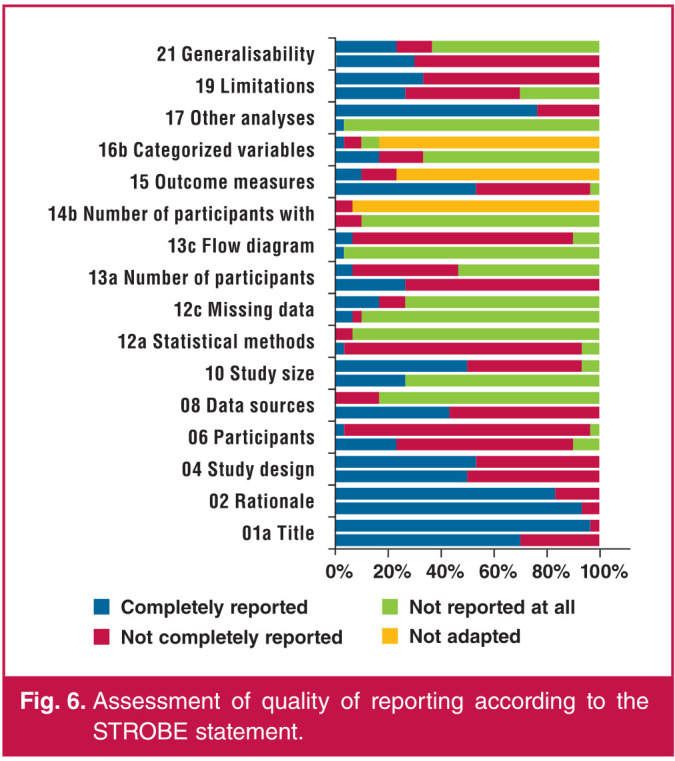
Assessment of quality of reporting according to the STROBE statement.

Concerning the quality of reporting, 32% (287/884) of the STROBE items were not reported at all. Of the 30 articles, on average, 32% (282/884) of items were completely reported and 36% (315/884) were not completely reported. Of the 30 articles, three^40^,^41^,^43^ had more than 60% of the STROBE items completely reported. Notable items that were not completely reported or not reported at all were with regard to methods, as detailed in Fig. 6, particularly those used to examine subgroups and interactions, flow diagram and the number of participants with missing data.

## Discussion

Our systematic review retrieved 30 observational studies reporting how antihypertensive medications were prescribed and assessing the influence of different antihypertensive medication strategies on BP control in SSA countries.

Overall, 21% of patients received monotherapy, 42.6% two-drug combinations and 26.6% three-drug combinations. Among all the strategies, RAS blockers were the most commonly prescribed BP-lowering drugs, followed by diuretics and CCB. In monotherapy, RAS blockers were the first class, and diuretics and CCB usage exceeded RAS blockers only in two-drug strategies. Monotherapy and two-drug combinations were well described. However, only 10 articles described antihypertensive drug strategies beyond triple combinations. BP control was highly variable from one country to another (range: 16.4–61.2%).

The description of patients’ cardiovascular risk factors or co-morbidities was heterogeneous and not precisely described. Diabetes mellitus was the most common cardiovascular risk factor reported. The other cardiovascular risk factors were scarcely described, especially obesity and dyslipidaemia. Moreover, the proportion of patients with associated cardiovascular risk factors varied widely across studies.

In Africa, 130.2 million people suffer from hypertension, a figure that is expected to reach 216.8 million by 2030.50 The highest prevalence of hypertension in the world is in SSA.^4^ In highincome countries, improvements in hypertension control have led to considerable reduction in overall morbidity and mortality rates over the last 50 years.^51^ Several international guidelines combining lifestyle modification and BP-lowering medications were elaborated on and contained specific recommendations for black adults living mostly in high-income countries outside of Africa.^6^ Those guidelines were mentioned in most of the articles in the review, however, it was difficult to conclude whether guidelines were sufficiently followed.

Lifestyle measures were only mentioned in a quarter of the articles. Surprisingly, RAS blockers were the most commonly prescribed BP-lowering drugs, even though CCB and diuretics are recommended in black African-Americans or African- Europeans, according to guidelines. It was impossible to know whether RAS blockers were prescribed in a valid indication because of the numerous non-reported patients’ co-morbidities. Therefore, diuretics should be considered, particularly due to their low cost.^25^ RAS blockers, CCB and diuretics were also the most frequent antihypertensive drug classes prescribed in France over the 2000–2015 period.^52^

The antihypertensive medication used in monotherapy or in combination appeared to be insufficiently effective regarding high proportions of patients with uncontrolled BP. BP control varied widely across studies and hypertension severity was underestimated. Adherence to medication was poorly evaluated, even though it is an important determinant in BP control.^53^ Many other underestimated physician- and patient-related factors may have contributed to poor BP control. These include (1) physician therapeutic inertia, as suggested by the low proportion of patients with combination therapy despite poor BP control,^54^ (2) high salt consumption by SSA patients, especially of middle age, such as in the reported populations,^55^ and (3) treatment affordability for patients.^56^

In the CREOLE randomised trial, where treatment were provided free of charge to patients, CCB appeared to be the most effective BP-lowering medicines.^57^ Treatment availability was also an important determinant of BP control but Rockers et al. and Geldsetzer et al. showed that without public health policies, BP control could not be achieved.^58^,^59^

Our review was complementary of the review by Seeley et al.^5^ We included only observational studies to provide data from real-world settings, whereas the review by Seeley et al. included controlled trials, therefore showing that CCB were the most effective agent to reduce BP in patients from SSA. This was not the picture we got from our analysis of observational studies reflecting more clinical practice in these countries.^5^

The discrepancy between the results may also be explained by the difference in countries studied. Seeley et al. included a smaller number of Nigerian studies and a larger number of South African studies. This difference may reflect a differential access to the different drug classes in SSA countries. It may also relate to the perceived confidence to the quality, efficacy and safety of the most prescribed antihypertensive drug classes by treating physicians. We believe that this difference does not reflect on aggressive marketing campaigns by pharmaceutical industries since all the drug classes are now generic drugs.

English-speaking countries were the most represented countries in our review [Nigeria (n = 14), Ethiopia (n = 4), South Africa (n = 2) and Kenya (n = 2)]. The lack of publications from western Sub-Saharan countries suggests a geographical inequity in access to English publications since English is currently used among physicians. Of the 30 articles included, 29 (96.6%) had at least one author affiliated to an African institution, suggesting the involvement of African physicians is improving the management of hypertension in their respective countries. Authorship representation and positioning provide one method of measuring African participation and leadership in research, as well as the possibility for Africans to negotiate decision-making in collaborative research performed in their countries.^60^

Only one of the 30 studies included had an overall risk of bias rating of good. This may show a lack of quality in the study methodology. Therefore, the quality of reporting was heterogeneous and items regarding the methods, particularly, were not completely reported. No study included in the review was multinational, and only three studies were multicentre. The studies included may not be representative enough of antihypertensive strategies employed in SSA and there is a significant publication barrier for resource-constrained institutions.^5^

## Limitations

This systematic review had some limitations. Data reporting was incomplete because we did not contact the authors. The reference list of articles included was not scanned and unpublished literature as well as grey literature were not reviewed. We restricted our search to begin in 2003. This choice was based on convenience as it was the year of publication of guidelines by ethnicity (black/non-black) in major guidelines of the worldwide society (American and European society). Our research strategy excluded articles where studies were not conducted exclusively in a SSA country. In these large multinational studies, details of data by African countries were not available.^61^,^62^

In this study, Nigerian patients were over represented: 40.7% compared to other countries. Indeed, some explanations could be discussed, such as Nigeria is an English-speaking country that publishes many studies in this field. Two studies included in the systematic review were from the same team (Ysuff et al.^18^) and were published closely. We could not exclude a possible overlap in terms of enrolled patients in these two studies, even if numbers and proportions were different. The settings of the majority of studies were in tertiary-care centres.

Even though ‘special populations’ (secondary hypertensives) were excluded, the lack of data regarding the lower level of care does not allow for drawing conclusions regarding the therapeutic strategies most commonly used, given that a large proportion of patients receive treatment for hypertension at a lower level of care. The populations at tertiary level are most likely not the same as those accessing lower levels, and prescription patterns might be very different (in various countries some classes of drugs are not available at primary levels of care). Moreover, they assessed mostly urban areas and this is hardly generalisable to all of SSA.

We excluded studies where the principal inclusion criterion was stroke. The burden of stroke is high in SSA and few data are available on long-term mortality. However, hypertension remains the most important modifiable risk factor for stroke in Africa. A new study focusing on stroke and hypertension is necessary.

Adherence to medication was assessed in only three studies and could be an important element in explaining poor BP control despite a majority of patients being on two and three agents. We possibly underestimated interactions between drug regimens and co-morbidities that may contribute to antihypertensive drug choice, drug dosage and potential resistant hypertension.

## Conclusion

Our review summarises antihypertensive strategies employed in SSA to achieve BP control. Our analysis provides the opportunity to extend knowledge on the effectiveness of antihypertensive strategies in SSA. Our systematic review showed that there are likely too many patients on monotherapy. Multicentre and multinational studies in rural and urban cities are needed to ensure international guidelines actually do improve outcomes in low- and middle-income countries. Studies should be designed with attention to methodology to collect generalisable data to develop hypertension public health policies.

**Table:** Key messages

Few comprehensive syntheses of pharmacotherapy used to manage hypertension in SSA are available. Too few studies had a good overall rating risk of bias, and there is a significant publication barrier for resource-constrained institutions. Multicentre and multinational studies in rural and urban cities are needed, and studies should be designed with attention to methodology.
